# c-Jun Contributes to Transcriptional Control of GNA12 Expression in Prostate Cancer Cells

**DOI:** 10.3390/molecules22040612

**Published:** 2017-04-10

**Authors:** Udhaya Kumari Udayappan, Patrick J. Casey

**Affiliations:** 1 Program in Cancer and Stem Cell Biology, Duke-NUS Medical School, 8 College Road, Singapore 169857, Singapore; gmsuuk@nus.edu.sg; 2 Department of Pharmacology and Cancer Biology, Duke University Medical Center, Durham, NC 27710, USA

**Keywords:** G12 proteins, transcription, metastasis, invasion

## Abstract

GNA12 is the α subunit of a heterotrimeric G protein that possesses oncogenic potential. Activated GNA12 also promotes prostate and breast cancer cell invasion in vitro and in vivo, and its expression is up-regulated in many tumors, particularly metastatic tissues. In this study, we explored the control of expression of GNA12 in prostate cancer cells. Initial studies on LnCAP (low metastatic potential, containing low levels of GNA12) and PC3 (high metastatic potential, containing high GNA12 levels) cells revealed that GNA12 mRNA levels correlated with protein levels, suggesting control at the transcriptional level. To identify potential factors controlling GNA12 transcription, we cloned the upstream 5′ regulatory region of the human *GNA12* gene and examined its activity using reporter assays. Deletion analysis revealed the highest level of promoter activity in a 784 bp region, and subsequent in silico analysis indicated the presence of transcription factor binding sites for C/EBP (CCAAT/enhancer binding protein), CREB1 (cAMP-response-element-binding protein 1), and c-Jun in this minimal element for transcriptional control. A small interfering RNA (siRNA) knockdown approach revealed that silencing of c-Jun expression significantly reduced GNA12 5′ regulatory region reporter activity. In addition, chromatin immunoprecipitation assays confirmed that c-Jun binds to the GNA12 5′ regulatory region in PC3 cells. Silencing of c-Jun expression reduced mRNA and protein levels of GNA12, but not the closely-related GNA13, in prostate cancer cells. Understanding the mechanisms by which GNA12 expression is controlled may aid in the development of therapies that target key elements responsible for GNA12-mediated tumor progression.

## 1. Introduction

Heterotrimeric G proteins transmit a wide variety of extracellular signals to effector molecules within the cell. Heterotrimeric G proteins, consist of a guanine nucleotide-binding α-subunit, and β- and γ-subunits, are classified into four subfamilies based on the sequence similarity of their alpha subunits. The G12 subfamily, which is comprised of the α-subunits Gα12 and Gα13, encoded by *GNA12* and *GNA13* genes, has been implicated in cellular processes such as Rho dependent cytoskeletal changes, cell polarity, cell growth and tumorigenesis and cell adhesion, migration and invasion [1,2,3]. 

In addition to studies linking G12-associated processes with tumorigenesis [4,5,6,7,8], GNA12 signaling induces a striking increase in cancer cell invasion in vitro [4,5,7], and inhibition of GNA12 signaling significantly reduces breast cancer metastasis in vivo [4,5,6]. Interestingly, the enhanced signaling of GNA12 that occurs during tumor progression appears to be due to enhanced expression of the protein rather than to mutational activation. Therefore, it is considered important to understand the control of GNA12 expression; such an understanding could shed light into its role in cancer.

Expression of a protein can be controlled through a variety of transcriptional, and/or post-transcriptional processes. In this regard, GNA12 signaling has been linked in several studies to the phosphorylation of c-Jun [6,9,10,11] a member of the Activator protein-1 (AP-1) family of transcription factors. AP-1 can be activated by a variety of extracellular stimuli [12], and the genes it controls have been implicated in a wide range of cellular processes, including cell proliferation, survival and differentiation. 

In the present study, we describe characterization of the GNA12 5′ regulatory region, and show it to be a major contributor to control of GNA12 expression in PC3 cells. This region was found to contain a c-Jun transcription factor binding site, and we demonstrate the high expression of GNA12 in PC3 cells is at least in part due to activity of the c-Jun transcription factor, providing a mechanism for linking GNA12 expression to potent oncogenic signaling pathways.

## 2. Results

### 2.1. Correlation of GNA12 mRNA and Protein Levels in Prostate Cancer Cell Lines

**Figure 1:**
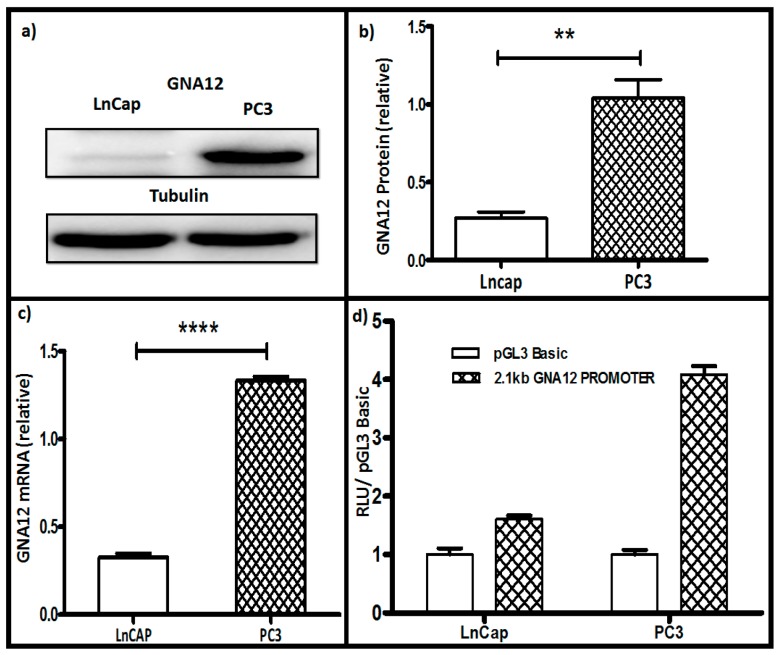
GNA12 mRNA and protein levels, and pGL3 Basic GNA12 reporter activity, in LnCap and PC3 prostate cancer cell lines. LnCap and PC3 cells were seeded in 6-well plates, after 24 h the cells were harvested and the total protein and total RNA were collected from the cells. (**a**) GNA12 protein levels determined by immunoblot, tubulin levels were assessed as an internal control. (**b**) Quantitation of data for (**a**). (**c**) Quantitative PCR (qPCR) analysis of GNA12 mRNA levels relative to those of glyceraldehyde 3-phosphate dehydrogenase (GAPDH) mRNA in the indicated cell lines. (**d**) Reporter activity of the full-length 2.144 kb GNA12-pGL3 Basic construct that was co-transfected with a renilla construct (as the internal control). Cells were harvested 48 h after transfection and luciferase levels determined. See Materials and Methods for further details. For panels **b**–**d**, data represent the mean and standard deviation of triplicate measurements from a single experiment that has been repeated three times with similar results. Significance was determined by *t*-test and is noted as ** *p* < 0.0017 and **** *p* < 0.0001.

Several studies have reported that GNA12 levels are highly up-regulated in cancers, with prostate cancer being among the first reported [4,5]. To explore the mechanism of GNA12 up-regulation in cancers, we chose to start with well-characterized prostate cancer cell lines. As shown in Figure 1a,b, the poorly-aggressive prostate cancer cell line, LnCAP (low metastatic prostate cancer cells), showed much lower levels of GNA12 protein than the much more aggressive PC3 line. This difference extended to GNA12 mRNA levels in these two cell lines, with PC3 cells showing almost five times the level of mRNA than the LnCAP cells (Figure 1c). These data suggested that GNA12 levels in the prostate cancer cells lines are controlled at the transcriptional level.

To begin to dissect the mechanisms of transcriptional regulation of GNA12, luciferase reporter constructs were generated to evaluate GNA12 5′ regulatory region reporter activity. The 2144 bp sequence from −1835 bp to +309 bp of the 5′ regulatory region of human GNA12 was cloned into the pGL3 Basic vector to create pGL3 Basic-GNA12. This construct was then co-transfected with renilla (as an internal control) into LnCap and PC3 cells, which were then analyzed by the Dual luciferase assay. As shown in Figure 1d, the activity of the reported construct in the two cell lines correlated well with GNA12 mRNA and protein levels, being roughly 3-fold higher in PC3 cells as compared with LnCAP GNA12 5′ regulatory region reporter activity.

### 2.2. Identification of c-Jun as a Transcription Factor Impacting GNA12 Expression

**Figure 2:**
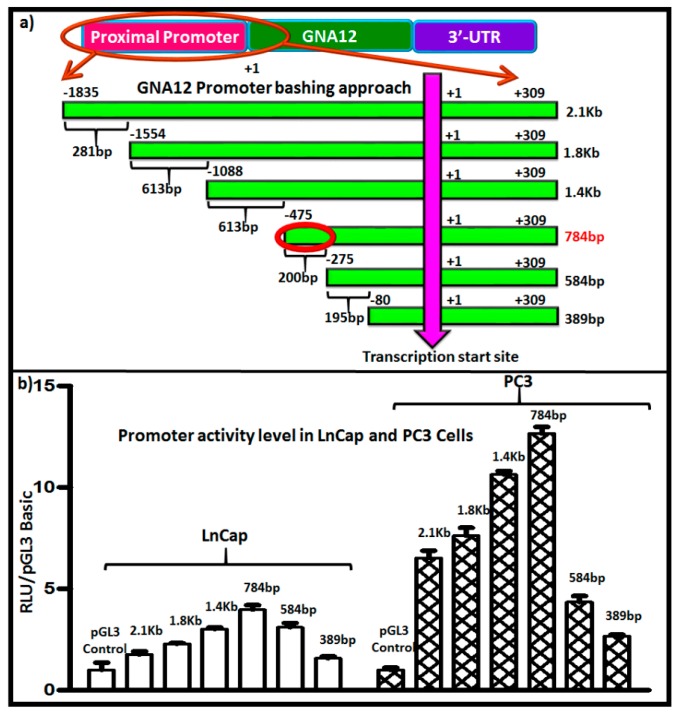
Identification of a 5′ regulatory region for GNA12. (**a**) The strategy for the deletion construct (5′ deletion) is described in the Materials and Methods section. (**b**) The prostate cancer cell lines LnCap and PC3 cells were plated into 24-well tissue culture dishes at 6 × 10^4^ cells/well 24 h before transfection. pGL3 Basic-GNA12 (luciferase) plasmids constructs were co-transfected with pRL Tk (renilla). Luciferase assays were performed 48 h after transfection using a Dual-Luciferase Reporter Assay System (Promega, Madison, WI, USA). Reporter activity was calculated using luciferase values normalized to renilla values (luciferase/renilla). See Materials and Methods for further details. For panel (**b**), data represent the mean and standard deviation of triplicate measurements from a single experiment that has been repeated three times with similar results.

To identify regulatory sequences that could be important in transcriptional control of the *GNA12* gene, we next created a series of 5′-deletion constructs of the full-length GNA12-5′ regulatory region and analyzed each for its ability to drive the expression of luciferase in LnCap and PC3 cells. The constructs are shown schematically in Figure 2a. Analysis of the 5′-deletion constructs revealed a distinct pattern of functional activity in the transfected cells (Figure 2b). The data indicate that the core 5′ regulatory region elements that drive maximal promoter activity are within the sequence spanning −275 bp to −475 bp upstream of the transcription initiation site, as further deletions displayed a marked loss of promoter activity (Figure 2b).

To determine whether this pattern of 5′ regulatory region activity was specific to the PC3 cells, the experiments were repeated in another prostate cancer cell line, DU145, and the same pattern of activity was observed (Supplementary Materials Figure S4a). As expected, GNA12 5′ regulatory region reporter activity for all constructs was much higher in PC3 and DU145 cells than in LnCap cells (Figure 2b). A similar pattern of activity of the 5′ regulatory region reporter constructs was also observed in the gastric cancer cell lines MKN7 (which contain low GNA12 levels) and YCC18 (which contain high GNA12 level) (data not shown).

**Figure 3:**
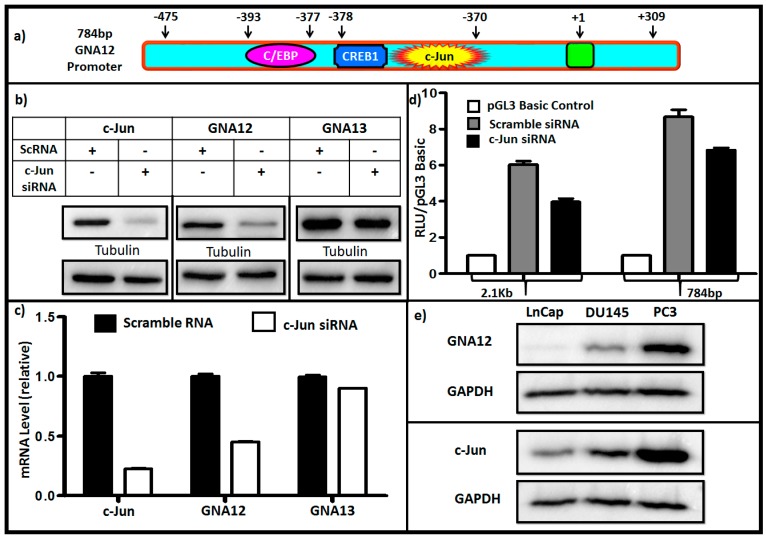
c-Jun knockdown suppresses GNA12 expression and 5′ regulatory region reporter activity. (**a**) Schematic description of transcription factor binding sites in the GNA12 5′ regulatory region. (**b**,**c**) PC3 cells were seeded 24 h prior to transfection. The cells were transfected with scramble control small interfering RNA (siRNA) or c-Jun siRNA as indicated and incubated for 48 h, whereupon RNA and protein were collected and analyzed by qPCR and western blotting for the indicated proteins. (**d**) pGL3 Basic-GNA12 constructs (luciferase) and pRL Tk (renilla) were co-transfected with scramble siRNA or c-Jun siRNA as indicated. Luciferase assays were performed 48 h after transfection using a Dual-Luciferase Reporter Assay. Reporter activity was calculated using luciferase values normalized to renilla values (luciferase/renilla). (**e**) LnCap, DU145 and PC3 cells were seeded in the 6-well format, after 24 h total protein was extracted and the endogenous levels of c-Jun and GNA12 assessed by immunoblot. See Materials and Methods for further details. For panels **c** and **d**, data represent the mean and standard deviation of triplicate measurements from a single experiment that has been repeated three times with similar results.

We next analyzed the 5′ region upstream of the transcription start site of GNA12 using the MatrixCatch v2.7 transcription factor finding [13]. A number of putative transcription factor binding sites were identified in the region from −275 to −475 bp that was responsible for maximal activity of the GNA12 5′ regulatory region reporter constructs (Figure 3a). The transcription factor-binding elements that were present in the 200 bp sequence included C/EBP (CCAAT/enhancer binding protein), CREB1 (cAMP-response-element-binding protein 1) and c-Jun. Binding sites for the transcription factor HMGA1 (High mobility group AT-hook 1) appear in the full length 5′ regulatory region (2144 bp) ten times and that for FOXA1 (Forkhead box protein A1) three times. We used RNAi to knockdown these transcription factors individually, and assessed the impact on GNA12 protein and mRNA levels in PC3 cells (Supplementary Materials Figures S1 and S2). Only the silencing of c-Jun (DNA binding sequence GAACCTCAG) resulted in a substantial reduction of GNA12 expression in the cells (Figure 3c). Importantly, GNA13 protein and mRNA levels were unaffected by c-Jun knockdown. A significant impact of c-Jun knockdown was also observed on the activities of both the full-length and the 784 bp GNA12 5′ regulatory region reporter constructs in PC3 cells (Figure 3d). Furthermore, deletion of c-Jun binding site from the 784 bp reporter construct resulted in the substantial reduction of reporter activity (Supplementary Materials Figure S3). 

**Figure 4:**
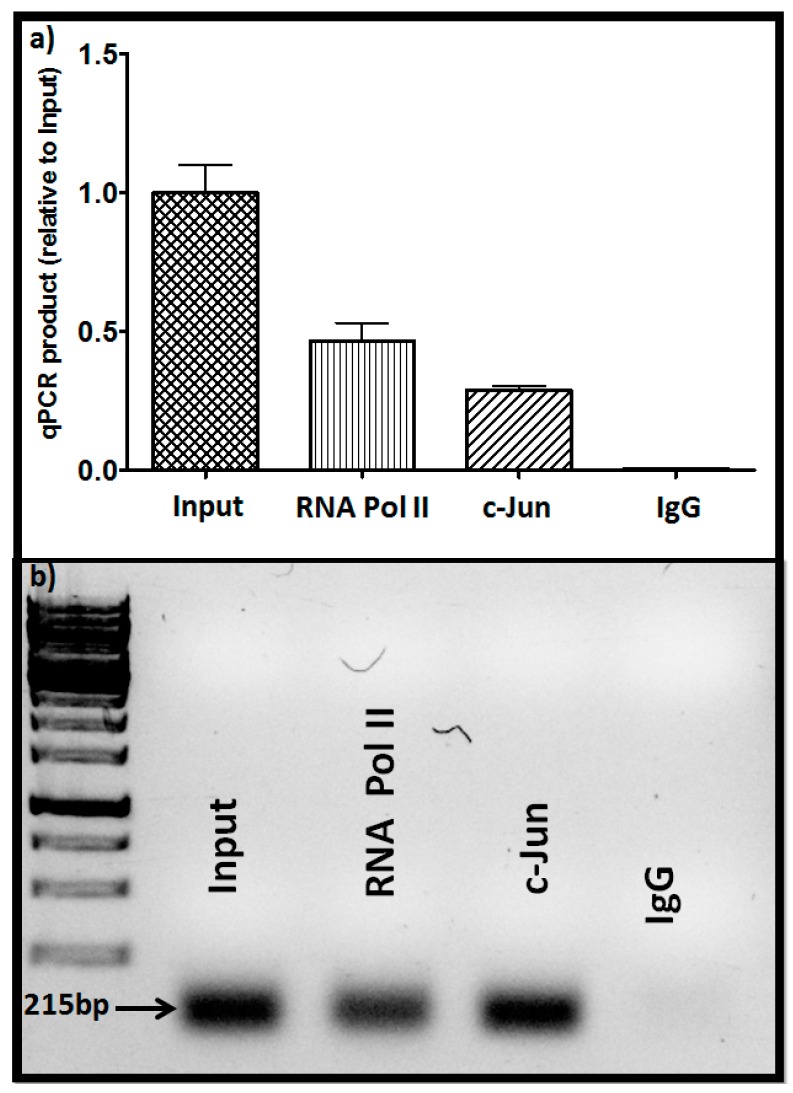
c-Jun binds to the GNA12-5′ regulatory region. (**a**) Identification of protein binding to the GNA12-5′ regulatory region by chromatin immunoprecipitation (ChIP) assays. Antibodies to c-Jun, RNA Pol II, and negative immunoglobulin G (IgG) were used to immunoprecipitate protein/DNA complexes from the sonicated lysates of PC3 cells. After reversing the cross-linking, DNA was precipitated and qPCR was performed using primers to amplify the −469/−254, 5′ regulatory region DNA (215 bp), after PCR amplification; (**b**) DNA gel analysis of the amplified PCR products. See Materials and Methods for further details. For panel **a**, data represent the mean and standard deviation of triplicate measurements from a single experiment that has been repeated three times with similar results.

Additionally, examination of the panel of prostate cancer cells from low metastatic to high metastatic (LnCap, DU145, PC3) showed parallel levels of c-Jun and GNA12; i.e., c-Jun level correlated with GNA12 level in the three cell lines (Figure 3e). Collectively, these results indicate that the c-Jun transcriptional factor plays an important role in the control of GNA12 transcription. As the data detailed above suggested a direct involvement of c-Jun in transcription of GNA12, we sought to determine whether recruitment of the transcription factor to the endogenous GNA12-5′ regulatory region during transcription could be detected. To this end, we performed standard chromatin immunoprecipitation (ChIP) assays in PC3 cells. Following crosslinking, immunoprecipitation of the chromatin with antibodies for c-Jun antibody, RNA polymerase II (as a positive control) and immunoglobulin G (IgG) (as a negative control) was undertaken. The immunoprecipitates were analyzed for the presence of a 215 bp region (−469 bp to −254 bp) of the GNA12-5′ regulatory region that contains the identified c-Jun binding site (−378 bp to −370 bp). Following amplification by qPCR, the 215 bp DNA fragment was clearly detected in the precipitates with the c-Jun and RNA polymerase II antibodies, but not the control IgG antibody (Figure 4). These results indicate that endogenous c-Jun can be bound to the region between −469 bp to −254 bp of the GNA12-5′ regulatory region in PC3 cells.

### 2.3. Silencing c-Jun Impacts GNA12-Dependent PC3 Cell Invasion

**Figure 5:**
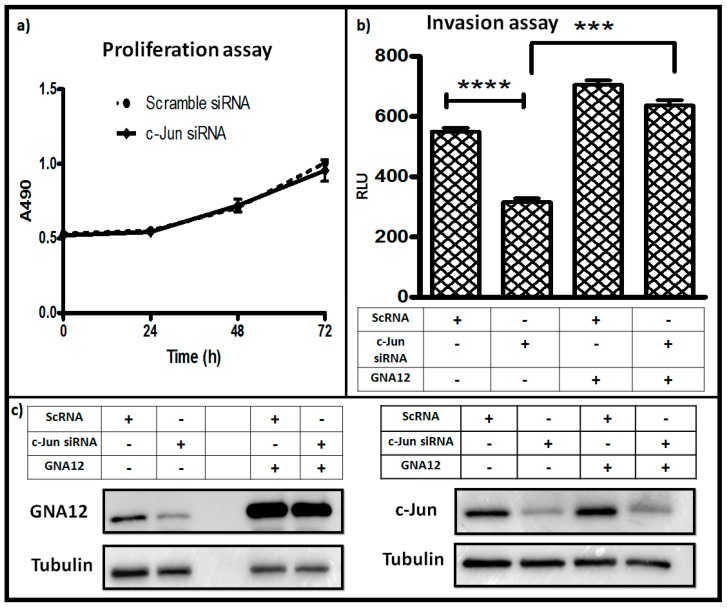
c-Jun knockdown impairs invasion of PC3 cells: (**a**) PC3 cells were seeded in the 24-well plate. After 24 h, cells were transfected with either scramble control or c-Jun siRNA and incubated for additional 24 h, 48 h or 72 h, as indicated. Proliferation of cells was determined by MTT (3-(4,5-Dimethylthiazol-2-yl)-2,5-diphenyltetrazolium bromide) assay. (**b**) PC3 cells were seeded in 10-cm dishes (Corning, New York, NY, USA) 24 h prior transfection. The cells were transfected with the indicated siRNAs and GNA12 plasmid and incubated an additional 24 h, then equally seeded in transwell chamber and incubated an additional 24 h. The cells were collected by trypinization and cell viability determined with CellTiter-Glo (Promega, Madison, WI, USA). (**c**) Immunoblot analysis of GNA12 (**left**) and c-Jun (**right**) levels in the samples from the experiment in panel **b**. See Materials and Methods for further details. For panels **a** and **b**, data represent the mean and standard deviation of triplicate measurements from a single experiment that has been repeated twice (panel **a**) or three times (panel **b**) with similar results. Significance was determined by *t*-test and is noted as *** *p* < 0.0001 and **** *p* < 0.0001.

Suppression of function of G12 proteins has been shown to negatively impact the ability of PC3 cells to invade in standard Matrigel assays [5]. Since silencing of c-Jun activity negatively impacted GNA12 levels (Figure 3), we reasoned that c-Jun should be important for PC3 cell invasion in a GNA12-dependent fashion. To test this hypothesis, PC3 cells in which c-Jun expression had been silenced were assessed both for their proliferation in a standard assay as well as for their invasion through Matrigel. The results revealed that there was no impact of c-Jun silencing on proliferation of the PC3 cells (Figure 5a). However, knockdown of c-Jun expression markedly inhibited the ability of the cells to invade Matrigel in response to serum as the chemoattractant (Figure 5b). Importantly, ectopic expression of GNA12 in the cells in which c-Jun was silenced largely rescued their ability to invade, providing evidence that a major component of the impact of c-Jun was through its influence on GNA12 levels in the cells. Immunoblot analysis of the cells confirmed the impact of c-Jun knockdown on GNA12 level, and the recovery of these levels upon ectopic expression of the GNA12 construct (Figure 5c). We also assessed the impact of c-Jun silencing in DU145 cells, another moderately metastatic prostate cancer cell line. Again, minimal impact of c-Jun silencing on proliferation, but a significant impact on cell invasion, was observed (Supplementary Materials Figure S4b,c). However, the ability of enforced expression of GNA12 to rescue the invasion phenotype of c-Jun silencing was greatly diminished compared to PC3 cells, suggesting a cell type-specificity to this result. Taken together, our data clearly indicate that c-Jun is an important control element for GNA12 expression in prostate cancer cells, and confirm a critical role for GNA12 in PC3 cell invasion.

## 3. Discussion

The discovery of GNA12 as a transforming oncogene provided the first evidence that enhanced expression of the wild-type protein alone could promote tumorigenesis [14]. This finding, along with recent studies implicating G12 proteins in cancer cell invasion and tumor progression [1,2,8,15], underscores the importance of elucidating the mechanisms through which levels of both GNA12 and its cousin GNA13 are controlled in cancer cells. For GNA13, post-transcriptional mechanisms involving specific microRNAs seem to be most important in this regard [16,17]. The current study suggest things are different for GNA12 and that, at least in the case of prostate cancer cells, the major point of control is at the transcriptional level.

Signaling through GNA12 had been previously shown to impact the phosphorylation of c-Jun [6,9] a major transcription factor involved in many aspects of cell regulation [18,19,20,21,22]. In the current study, we found that c-Jun is a major contributor to GNA12 transcription. Following up on the observation that mRNA and protein levels of GNA12 correlated in LnCap (low GNA12, low metastatic potential) and PC3 (high GNA12, high metastatic potential) cell lines, we examined the activity of GNA12-5′ regulatory region reporter constructs in the two lines. GNA12-5′ regulatory region reporter activity also paralleled the mRNA and protein levels, and dissection of the 5′ regulatory region led to the identification of c-Jun as directly targeting the element and as a positive regulator of GNA12 transcription in prostate cancer cells. Further, RNAi-mediated knockdown of c-Jun resulted in decreased GNA12-5′ regulatory region reporter activity, and mRNA and protein levels of GNA12, in both PC3 and DU145 cells. ChIP assays confirmed that endogenous c-Jun directly binds to the GNA12 5′ regulatory region in cells. Interestingly, while cell viability was not affected by c-Jun knockdown, PC3 cell invasion was markedly reduced in a fashion that could be rescued by ectopic expression of GNA12. These results demonstrate that c-Jun plays a critical role in *GNA12* gene expression and cell invasion in prostate cancer cells.

In summary, the current studies provide evidence showing that c-Jun directly binds to a consensus binding sequence within the GNA12-5′ regulatory region, thereby regulating GNA12 transcription. These findings reveal a novel mechanism for regulation of GNA12 expression, and provide an additional functional link between the GNA12 and c-Jun that contributes to the impact of this G protein on oncogenic processes (Figure 5). Understanding the mechanisms by which GNA12 expression is upregulated in tumor progression may help in the development of drugs that target key elements responsible for metastasis and cancer progression.

## 4. Materials and Methods

### 4.1. Cell Lines and Reagents

The human prostate cancer cell lines LnCap, DU145 and PC3 cell lines (obtained from the Duke Cell Culture Facility, Duke University, Durham, NC, USA) were maintained in RPMI (Roswell Park Memorial Institute Medium) with 10% fetal bovine serum (Invitrogen, Waltham, MA, USA). The c-Jun siRNAs (SI00034678 and SI00300580), non-targeting siRNA control, and c-Jun primer were obtained from Qiagen (Hilden, Germany). The Dual Luciferase Reporter Assay reagents were from Promega (Madison, WI, USA), and Jet prime reagent was from Polyplus (Illkirch, France). Matrigel was obtained from BD Biosciences (San Jose, CA, USA)). c-Jun Chip-grade antibody for ChIP assay was from Abcam (Cambridge, UK), c-Jun antibody for western blot was from Cell Signaling Technology (Danvers, MA, USA) the GNA12 antibody was from Gentex (Irvine, CA, USA), and GNA13 antibody was from Calbiochem (San Diego, CA, USA), α-Tubulin was purchased from Sigma (St. Louis, MO, USA), protease inhibitors were purchased from Roche Applied Science (Penzberg, Germany).

### 4.2. GNA12-5′ Regulatory Region Reporter Construct Cloning Strategies

A 2144 bp sequence consisting of −1835 bp to +309 bp of the promoter and 5′-untranslated region of the human GNA12 promoter gene was generated by PCR amplification of human genomic DNA that was isolated from HEK 293 (human embryonic kidney cells) using a DNeasy Blood & Tissue Kit (Qiagen) according to the manufacturer’s protocol. PCR was conducted using the isolated genomic DNA as template and dedicated primers from the *GNA12* gene containing Kpn1 and XhoI sites incorporated into the forward and reverse primers, respectively. The reaction was run for 35 cycles in the presence of 200 μm dNTP, 5% DMSO, 10% Betaine, and 0.02 U/μL of iProof DNA Polymerase (Bio-Rad, Hercules, CA, USA). PCR cycling condition were as follow: Initial denaturation 98 °C for 30 s; denaturation 98 °C for 10 s; annealing 55 °C–65 °C for 30 s; extension at 72 °C for 75 s for 35 cycles and final extension 72 °C for 10 min. The amplified fragment was cloned upstream of the luciferase reporter pGL3 basic vector (Promega) after digestion with Kpn1 High Fidelity and XhoI. Using the above sequence as template, subsequent deletion constructs starting at −1554 bp, −1088 bp, −475 bp, −275 bp and −80 bp with a common 3′-end at +309 bp were generated in the pGL3 basic vector. Primers used for cloning and generating the deletion mutants are listed in Supplementary Materials Table S1. All the 5′ regulatory region reporter fragments were sequenced and verified.

### 4.3. Site Directed Mutagenesis

Within the pGL3 Basic GNA12 784 bp reporter construct, the c-Jun binding site (GAACCTCAG) were deleted using a QuikChange II XL site-directed mutagenesis kit according to the manufacturer’s protocol (Agilent Technology, Santa Clara, CA, USA) using the primers listed in Supplementary Materials Table S1. The c-Jun binding site deletion construct was verified by DNA sequencing.

### 4.4. Luciferase Reporter Assay

The prostate cancer cell lines LnCap and PC3 cells were plated into 24-well tissue culture dishes at 6 × 10^4^ cells/well and incubated 24 h before transfection. Transfections were performed in quadruplicate according to the manufacturer’s protocol using Jet prime reagent (Polyplus) with 0.5 μg reporter of the pGL3 Basic-GNA12 (luciferase) construct together with 100 ng of pRL Tk (renilla). Luciferase assays were performed 48 h after transfection using a Dual-Luciferase Reporter Assay System (Promega). Luciferase and renilla activities were read on a Tecan microplate reader (Männedorf, Switzerland). Luciferase activity was normalized to renilla readings in each well. All experiments were performed in quadruplicate and repeated three times to ensure reproducibility.

### 4.5. Bioinformatics Analysis

The sequence of the 2144 bp 5′ regulatory region of GNA12 was obtained from the Cold Spring Harbor Laboratory Transcriptional Regulatory Element Database (https://cb.utdallas.edu/cgi-bin/TRED/tred.cgi?process=home). Identification of putative transcription factor binding sites in the 5′ regulatory region of GNA12 was carried out using MatrixCatch software v2.7 [13].

### 4.6. RNA Extraction Quantitative Real-Time Reverse Transcription-PCR (qPCR)

Total RNA was purified using the RNeasy Kit (Qiagen) according to the manufacturer’s instructions and treated with DNase I minimize genomic DNA contamination. For reverse transcription, an input of 2 μg of total RNA was used for the first strand iScript cDNA synthesis Kit (Bio-Rad) according to the manufacturer’s instructions. The newly synthesized cDNA was amplified by qPCR using the CFX96 TouchReal-Time PCR Detection System and the Syber Green qPCR Kit (Bio-Rad). The amount of target transcript was normalized to the amount of GAPDH transcript. Primer sequences are shown in Supplementary Materials Table S1.

### 4.7. Immunoblot Analysis

Cells were washed twice with cold PBS and lysed using cell extraction buffer 20 mM HEPES, pH 8.0, 1 mM EDTA, 3 mM dithiothreitol, 10 mM MgSO_4_, 150 mM NaCl, 1% Triton X-100. The protein concentration was quantified using the Pierce BCA Protein Assay kit (Thermo Scientific, Waltham, MA, USA). Protein samples (20–30 μg) were separated by 8.5% SDS-PAGE, transferred onto an Immun-Blot PVDF membrane (Bio-Rad) and blotted with GNA12 antibody, GNA13 antibody, Tubulin, and c-Jun antibody, and visualized by Western Lightning-ECL (Millipore, Billerica, MA, USA).

### 4.8. Proliferation and Invasion Assays

LnCap or PC3 cells were seeded at 5 × 10^3^ cells/well of a 96-well plate and incubated at 37 °C in 5% CO_2_ for 24 h before transfection with control scramble RNA or c-Jun siRNA. Proliferation was determined using the CellTiter 96 Aqueous one (Promega) assay. Briefly, 10 μL of the reagent was added to each well and incubated at 37 °C for 1 h in a humidified, 5% CO_2_ atmosphere, whereupon the absorbance reading was taken at 490 nm (Tecan Micro plate reader). For invasion assays, PC3 cells were seed in 10 cm dishes and transfected with control scramble RNA or c-Jun siRNA. After 24 h, the cells were trypsinized, washed, resuspended in serum-free RPMI-1640, and 1 × 10^5^ cells plated in the upper chamber of 8-μm pore transwell chambers coated with 20 μg Matrigel. The lower chamber was filled with RPMI-1640 supplemented with 10% FBS as a chemoattractant. Following incubation at 37 °C for 24 h, non-migrated cells on the upper surface of the insert membrane were removed with a cotton swab. Cells on the lower side of membrane were removed by washing with PBS, and the CellTiter-Glo Luminescent Cell Viability Assay (Promega) was used to determine the number of viable cells. All experiments were performed in triplicate and repeated three times.

### 4.9. ChIP Assay

PC3 cells were cross linked with 1% formaldehyde for 10 min at room temperature, followed by a quench with 0.2 M glycine for 10 min. The supernatant was removed and cells were scraped into ice-cold PBS and centrifuged 1000 *g* for 5 min at 4 °C. Cells were lysed and sonicated to obtain 200–500-bp chromatin fragments. The chromatin was immunoprecipitated by incubating with antibodies and Magnetic beads (Invitrogen, Waltham, MA, USA). After the elution, the purified DNA reverse cross linked and was used to analyse by the Quantitative PCR using CFX96 Touch Real-Time PCR Detection System (Bio-Rad) and the Syber Green qPCR Kits (Bio-Rad). The reaction mixture contained 5 μL of ChIP or input DNA (1:100 dilution), 1 μL of forward and 1 μL of reverse primer, and 10 μL of Syber green Mastermix (Bio-Rad) in a total volume of 20. Amplification cycles were: 95 °C for 15 min, then 40 cycles at 95 °C for 15 s, 55 °C for 30 s and 72 °C for 30 s, followed by melt curve 65 °C to 95 °C. Melting curve analysis revealed a single PCR product. After the qPCR the amplified the PCR product also analyzed by DNA gel. Primer sequences listed in the Supplementary Materials Table S1.

### 4.10. Statistical Analysis

The data for proliferation, invasion luciferase reporter activities in are presented as the mean ± standard deviation (SD). Data were analyzed using GraphPad Prism v4 (GraphPad Software Inc., La Jolla, CA, USA). Data were tabulated as mean ± SEM and compared by one- or two-way ANOVA as appropriate, followed by the relevant post-test to determine *p* values. A probability of *p* < 0.05 was considered significant, with *p* < 0.01 considered very significant.

## Supplementary Materials

The following are available online. Figure S1: Impact of CREB and CEBP Beta knockdown on mRNA and protein levels of GNA12, Figure S2: Impact of HMGA1 knockdown on mRNA and protein levels of GNA12, Figure S3: Deletion of c-Jun binding site impacts GNA12 5’ regulatory region reporter activity, Figure S4: (a) GNA12 5’ regulatory region reporter activity in DU145 cells. (b) Impact of c-Jun silencing on DU145 cell invasion. (c) Impact of c-Jun silencing on DU145 cell proliferation. Figure S5: Impact of c-Jun knockdown using a siRNA targeting the c-Jun 5’ UTR, Table S1: Primers used in the studies.

## Abbreviations

The following abbreviations are used in this manuscript:

RLU	Relative light units
GPCR	G protein-coupled receptor
JNK	c-Jun N-terminal kinase
