# Supplementary material for: c-Jun Contributes to Transcriptional Control of GNA12 Expression in Prostate Cancer Cells
Source: Molecules. 2017 Apr 10;22(4):612. doi: 10.3390/molecules22040612 (PMC6153990; doi:10.3390/molecules22040612)
Supplement: Supplementary file 1 [file molecules-22-00612-s001.pdf]

Supplementary Table 1. Primers used in the studies

Oligonucleotide	Sequence (5’ to 3’)	Purpose
-1835bp to +309bp Forward primer (2144 bp)	ATGCGGTACCAGCCTTCTGGGGAGTTTAGATCTGTGACTA	Promoter cloning
-1554bp to +309bp Forward primer (1863bp)	ATGCGGTACCGAATAAAACTGGATCCTCGTCTCTCACGTT	Promoter cloning
-1088bp to +309bp Forward primer (1397 bp)	ATGCGGTACCGCTCACGTGCTAAGTGTTTGGTAAGTTCAT	Promoter cloning
-475bp to +309bp Forward primer (784 bp)	ATGCGGTACCCACGTCTGATGTCCACTCAGAGCAATTAT	Promoter cloning
-275bp to +309bp Forward primer (584 bp)	ATGCGGTACCTTGGCGAGATGAGCCAATCG	Promoter cloning
-80bp to +309bp Forward primer (389 bp)	ATGCGGTACCTCGGCCCGGGCTCGCCCTCG	Promoter cloning
Common Reverse primer for promoter cloning	ATGCCTCGAGCTTGAGGATGTTGTCGAAGATGGTGTC	Promoter cloning
GNA12 Forward primer	TCTGGCATCAGGGAGGCTTTCA	Q PCR
GNA12 Reverse primer	TTCCCTTGGTGGCTTTCCTAGC	Q PCR
GAPDH Forward primer	GTCTCCTCTGACTTCAACAGCG	Q PCR
GAPDH Reverse primer	ACCACCCTGTTGCTGTAGCCAA	Q PCR
GNA13 Forward primer	TCCACCTTCCTGAAGCAGATGC	Q PCR
GNA13 Reverse primer	GCTTCTCTCGAGCATCAACCAG	Q PCR
Chip assay Forward primer	TGATGTCCACTCAGAGCAATTAT	Chip assay
Chip assay Reverse primer	CCCTTCGATTGGCTCATCTC	Chip assay
c-Jun site deletion sense primer	CCTCCCTTTCATTCTGAAAGAAACCTGGGCGTCCA	Site directed mutagenesis
c-Jun site deletion antisense primer	TGGACGCCCAGGTTTCTTTCAGAATGAAAGGGAGG	Site directed mutagenesis

Supplementary figure: 1

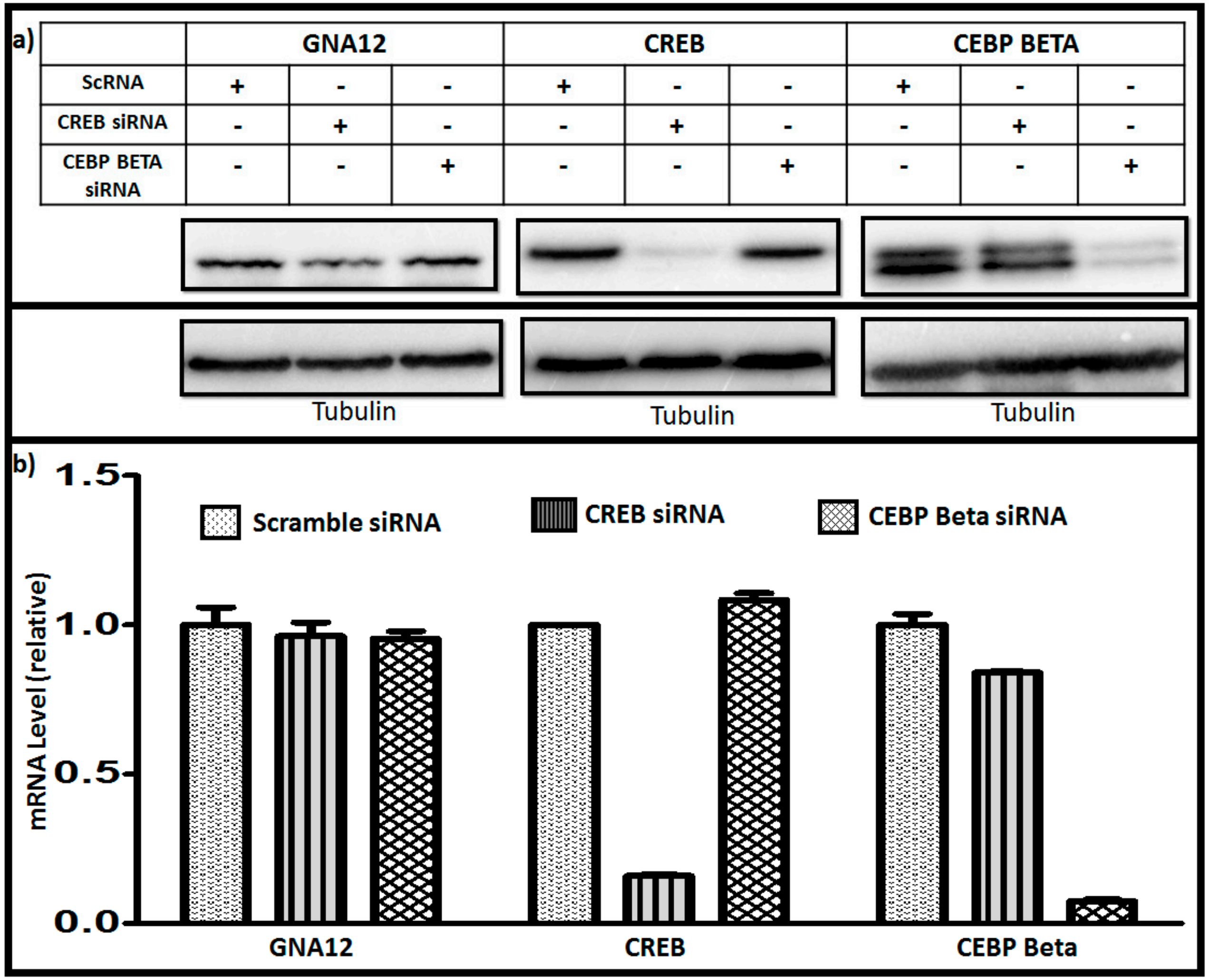

Supplementary Fig. 1: Impact of CREB and CEBP Beta knockdown on mRNA and protein levels of GNA12: PC3 cells were seeded 24 h prior to transfection. The cells transfected with control siRNA, or siRNA targeting CREB or CEBP Beta as indicated, and cultured an additional 48 h. a) Protein levels analyzed by western blotting. b) mRNA level analyzed by qPCR.

Supplementary figure: 2

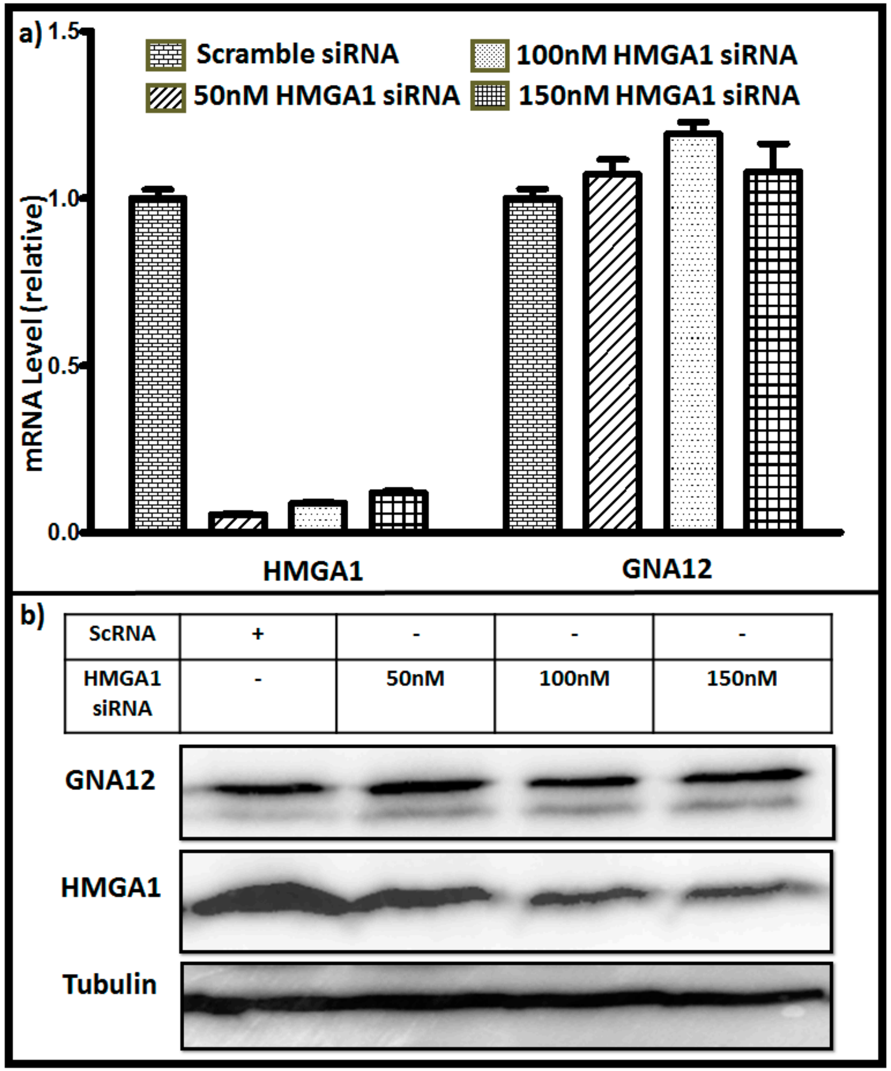

Supplementary Fig. 2: Impact of HMGA1 knockdown on mRNA and protein levels of GNA12: a) PC3 cells were seeded 24h prior to transfection. The cells transfected with Scramble control siRNA or HMGA1 siRNA and cultured an additional 48h. a) mRNA levels analyzed by qPCR. b) Protein levels analyzed by western blotting.

Supplementary figure: 3

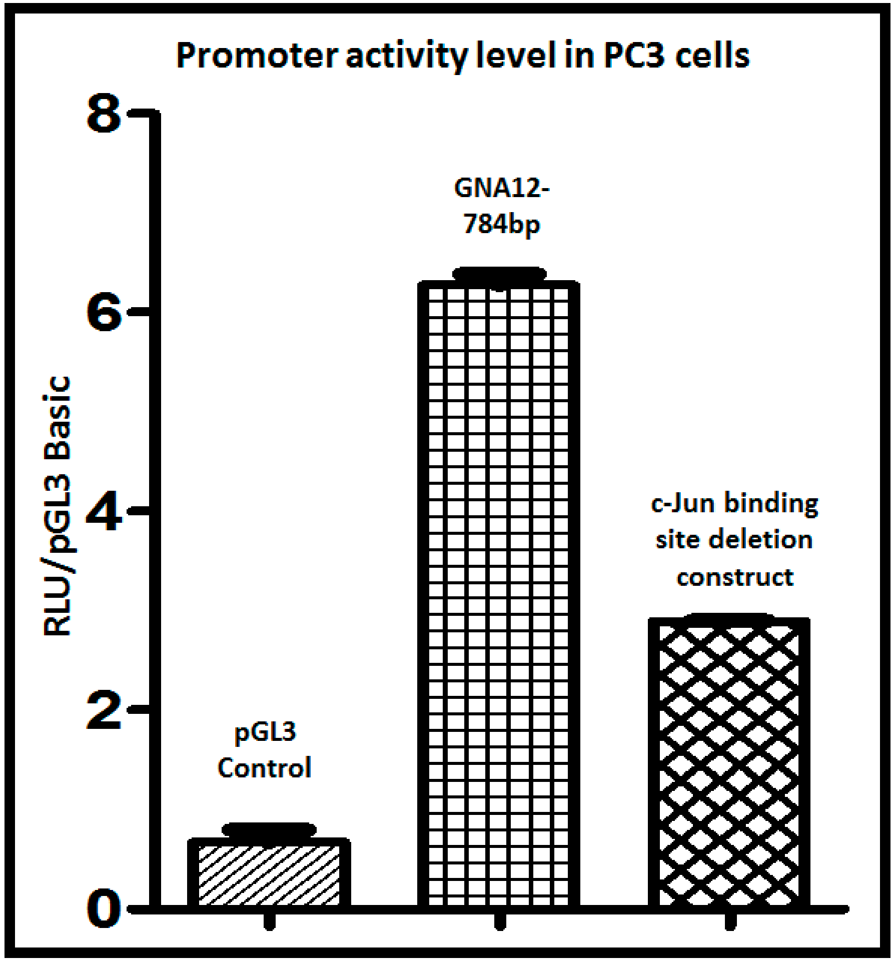

Supplementary Fig. 3: Deletion of c-Jun binding site impacts GNA12 5' regulatory region reporter activity: PC3 cells were seeded 24 h prior transfection, whereupon plasmids pGL3 Basic (control), as well as the reporter constructs containing the GNA12 784 bp 5' element or that containing the 784 bp element with the c-Jun binding site deletion, along with pRL Tk (Renilla), were co-transfected. After an additional 48 h, cells were harvested and luciferase level determined. Reporter activity was calculated using luciferase values normalized to renilla values (Luciferase/Renilla). See Materials and Methods for details.

Supplementary figure: 4

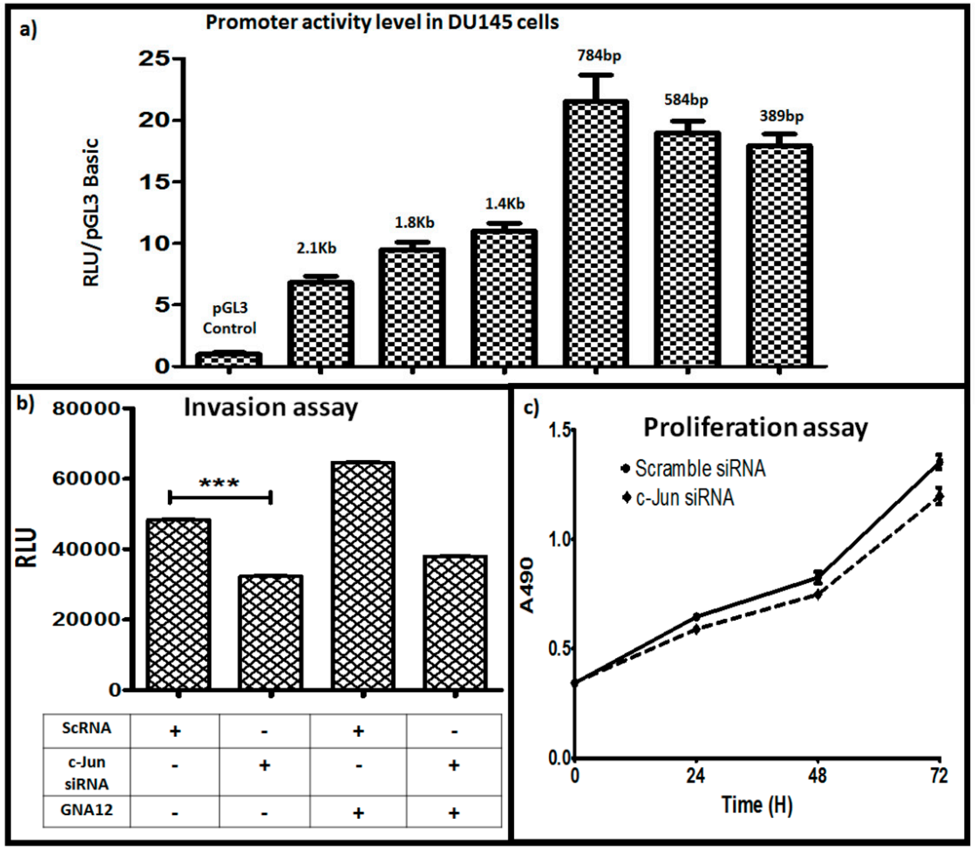

Supplementary Fig. 1: a) GNA12 5' regulatory region reporter activity in DU145 cells. Cells were seeded 24h prior transfection whereupon plasmids encoding pGL3 Basic-GNA12 (luciferase) and pRL Tk (Renilla) were co-transfected. After an additional 48 h, cells were harvested and luciferase level determined. Reporter activity was calculated using luciferase values normalized to renilla values (Luciferase/Renilla). b) Impact of c-Jun silencing on DU145 cell invasion. DU145 cells were seeded 24 h prior to transfection with the indicated siRNA and cultured an additional 24 h before being seeded in transwell chambers and left an additional 24h. The cells that passed the filter under the Matrigel were collected by trypinization and viable cells measured with CellTiter-Glo. c) Impact of c-Jun silencing on DU145 cell proliferation. DU145 cells were seeded 24 h prior transfection with either control siRNA or c-Jun siRNA and cultured for the indicated periods. Viable cells were measured by MTT assay.

Supplementary figure: 5

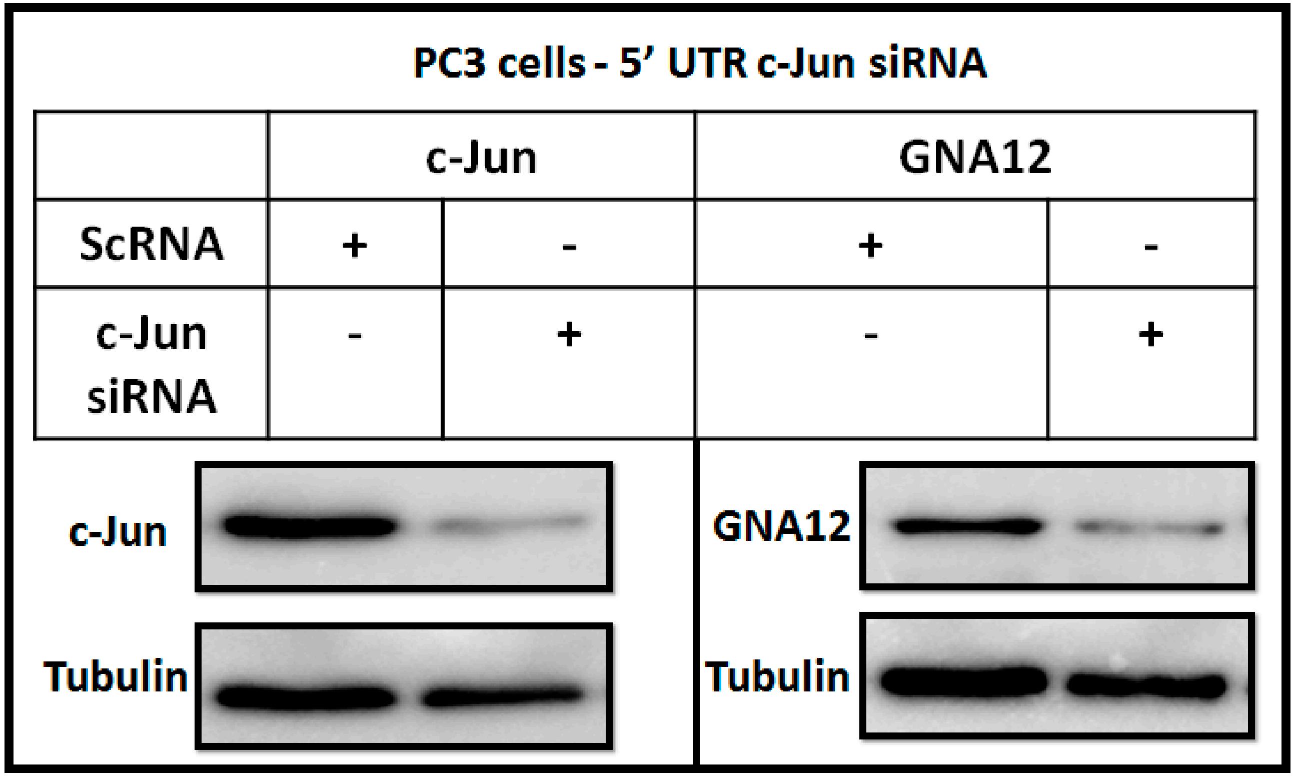

Supplementary Fig. 5: Impact of c-Jun knockdown using a siRNA targeting the c-Jun 5' UTR. PC3 cells were seeded 24 h prior to transfection with control siRNA or c-Jun siRNA and cultured and additional 48 h. The indicated proteins were analyzed by western blot.
